# Quality of life and epidemiological profile of male breast cancer treated at the university hospital of Casablanca, Morocco

**DOI:** 10.11604/pamj.2022.41.127.28319

**Published:** 2022-02-14

**Authors:** Majdouline El Fouhi, Bouchra Haddou Rahou, Abdelhalim Mesfioui, Abdellatif Benider

**Affiliations:** 1 Biology and Health Laboratory, Ibn Tofail University, Kenitra, Morocco,; 2 Research Department, Higher Institute of Nursing Professions and Technical Health, Rabat, Morocco,; 3 Mohammed VI Cancer Treatment Center, Ibn Rochd University Hospital, Casablanca, Morocco

**Keywords:** Quality of life, male, breast cancer, epidemiology

## Abstract

Nowadays, cancer is a huge public health challenge that needs for more advanced researches. Quality of life of patients with breast cancer is an important outcome. Data analyses are usually referred to female breast cancer studies and limited informations are available about male breast cancer. Our study is the first in our country to assess quality of life (QoL) in male patients affected by breast cancer. The purpose of this study is to investigate HRqol (health related quality of life) in male patients with breast cancer and clinico-pathological features at the university hospital of Casablanca, Morocco over a period of 6 years. This study involved 21 male subjects from 2012 to 2018. Required information were collected from the medical records of patients in the oncological center. We included demographic, clinical and pathological characteristics. HRqol was investigated using the European Organization for Research and Treatment of Cancer 30-Item QoL Questionnaire (EORTC QLQ-C30), version 3.0. Mean age of patients at enrollment was 67.3 years (SD=15.6, range=36-87 years), the average consultation delay was 17,7 months, the most common histologic finding was infiltrating ductal adenocarcinoma (20 patients, 95.3%). Progesterone and estrogen receptors were positives in 90.4% (19 patients) of cases, the most representative stage was stage III, the most common molecular phenotype was Luminal B (16 patients, 76.2%), modified radical mastectomy was the main surgical procedure. Adjuvant therapy was based on chemotherapy (100%), radiotherapy (76.2%), hormone therapy (90.5%). Ten patients (47.6%) had metastasis. A moderate overall quality of life was reported, with a mean of 50±21.73. The results showed a mean physical function score (54.60±27.85), positive emotional functioning (56.34±31.94) and good social functioning (75.39±17.96). In brief, regarding QOL in this population, it appears to be better than expected andQOL generally improves after treatment. As for prevention, public education should be oriented toward men at higher risk in order to reduce the time between onset of symptoms and consultation.

## Introduction

Male breast cancer accounts for around 1% of all breast cancers, and relatively little is known about its etiology. According to the World Health Organization (WHO, 2012), quality of life (QoL) is defined as individual perception of life, values, objectives, standards, and interests in the framework of culture. Quality of life is increasingly being used as a primary outcome measure in studies to evaluate the effectiveness of treatment [1]. Many factors positively or negatively affect quality of life. Tiredness, anxiety, concern for the future and the family, difficulties to meet basic demands and changes in body image worsen the quality of life of cancer patients. Social support, economic security and faith in recovery improve quality of life [2-4]. Quality of life has been described as the subjective evaluation of life as a whole or patient's appraisal and satisfaction with current level of functioning compared with what the patient perceives to be possible or ideal [5]. Understanding the effect of breast cancer and his treatment on patient's QoL has been a central clinical and research question. Studies have shown that breast cancer and its treatment processes affect the QoL of patients in the physical, psychological and social domain [6]. Few studies have examined quality of life with standardized instruments. We conducted this study of male subjects in Morocco because of the limited information on the QoL of breast cancer patients. The purpose of this study was to investigate health-related quality of life (HRQoL) and epidemiological profile of Moroccan male breast cancer patients at the university hospital of Casablanca Ibn Rochd.

## Methods

**Study design and data collection:** this study included 21 male patients over the period from 2012 to 2018. The required information were collected from the medical records of patients in the oncological center of the university hospital Ibn rochd. Inclusion criteria were male patients with localized, locally advanced or metastatic breast cancer. We excluded patients who didn´t initiate follow-up after initial diagnosis. All the diagnoses of breast cancer had preoperative histological confirmation. Demographic characteristics included: age at diagnosis, residence, marital status, social and economic level, employment status, social security. Clinical and pathological characteristics included: tumor size, axillary lymph node status, cancer´s stage, average diagnosis delay, SBR grade, histology, and hormone receptor expression, molecular phenotype, treatment and metastasis.

**Instruments and procedures and statistical analysis:** data were collected using a sociodemographic and clinical form: the European Organization for Research and Treatment of Cancer 30-Item QoL Questionnaire (EORTC QLQ-C30), version 3.0. This QoL measurement tool includes 30 questions; global health status/QoL questions, 15 multi-element functional scales (physical and emotional, cognitive and social functioning), 13 multi-element symptomatic scales. The different scale scores are between 0 and 100. An overall health status (HRLQ)score close to 100 indicates perfect health. Similarly, a functional scale score close to 100 represents a perfect capacity level. In contrast, a symptom scale score close to 100 represents a high symptom load [7, 8]. The EORTC QLQ-C-30 questionnaire was translated into Moroccan dialectal Arabic, adapted and validated for Moroccan context [9]. Interview technique was used as method to gather data while control checking, statistical analysis were performed using SPSS 21.

**Ethical considerations:** informed consent was obtained from all participants after a detailed explanation of the study.

## Results

A total of 21 male patients were included in the study, the sociodemographic characteristics are summarized in Table 1. Mean age was 67.3 years (SD=15.6, range=36-87 years), a total of 14 (66.6%) patients were from an urban background and 7 (34.4%) from a rural background, one case was reported having a family history, 18 patients (85.7%) were covered by social security (Table 1). The average consultation delay was 17.7 months [range= 0.5-48 months]. Most patients had infiltrating ductal carcinoma (20 patients, 95.3%). Left sided breast cancer was slightly preponderant (63.1%) and the most common molecular phenotype was Luminal B (16 patients, 76.2%). Clinically staged T3-T4 cancers were the most common types (53.8%), 61.9% had lymph node metastases (13 patients) in the histopathology specimen. The number of positive lymph nodes was in the range of 1-17; 90.4% (19 patients) of cases had progesterone and estrogen receptors positive cancer, 81% of patients underwent mastectomy, all patients received chemotherapy, 16 patients (76.2%) received radiation therapy, 19 patients (90.5%) of participants received adjuvant hormonal therapy and trastuzumab was given to 10 patients (47.6%), lung was the representative site of metastasis (54.5%) (Table 2).

**Table 1:** socio-demographical characteristics (N=21)

	Characteristics	N(%)
**Age**	**Age (mean±SD)**	**(67.3±15.6)**
**Residence**		
	Urban	14(66.6%)
	Rural	7(33.4%)
**Marital status**		
	Maried	16(76.2%)
	single	2(9.5%)
	widower	3(14.3%)
**Social and economic level**		
	low	13(62%)
	Medium	8(38%)
	high	-
**Employment status**		
	Active	9(42.8%)
	Inactive	12(57.2%)
**Social security**		
	Yes	18(85.7%)
	No	3(14.3%)

**Table 2:** clinico-pathological characteristics (N=21)

	Characteristics	N (%)	Mean	SD
Consultation delay	months	-	17.7	±21.4
Imaging	BIRADS	-	4	±1.4
**Histological type**				
	Invasive ductal	20(95.3%)	-	-
Tumor size(cm)				
	carcinoma	1(4.7)	-	-
**Lymph node status**				
	Invasive lobular carcinoma	-	3.4	±1.3
	metastasis	13(61.9%)		
	no	8(38.1%)	-	-
SBR grade	(mean ±SD)	-	2	±0.5
Hormone				
**Emboles vasculaire**				
	Positive	19(90.5%)	-	-
	negative	2(9.5%)		
	Yes	13(61.5%)	-	-
	-no	8(38.5%)		
Ki67	(mean ±SD)	-	29.3	±14.1
Cancer stage	(mean ±SD)		3	1.2
**Hormonal receptor**				
	Positive	19(90.5%)		
	Negative	2(9.5%)		
**Molecular phenotype**				
	-Luminal A	2(9.5%)	-	-
	-Luminal B	16(76.2%)	-	-
	-HER+	1(4.8%)	-	-
	-Basal like	2(9.5%)		
**Metastasis**				
	Yes	10(47.6%)	-	-
	no	11(52.4%)	-	-
**Traitement**				
	Chemotherapy	21(100%)	-	-
	Radiotherapy	16(76.2%)	-	-
	Hormone therapy	19(90.5%)	-	-
	herceptine	10(47.6%)	-	-

All patients received curative cancer therapy; moderate overall quality of life was reported, with an average of 50±21.73. The results showed mean physical function score (54.60±27.85), positive emotional functioning (56.34±31.94) and good social functioning (75.39±17.96). In our study, a mean of 61.9±31.34 showed fatigue, a mean of 43.88±36.15 experienced nausea/vomiting, a mean of 40.47±29.61 pain, a mean of 36.50±31.45 dyspnoea (), a mean of 41.26±37.86 insomnia, a mean of 46.03±34.11 appetite loss, 20.8% experienced constipation (44.44±79.11) and diarrhoea (28.57±42.53) (Table 2). Among the 21 patients, financial difficulties were declared in an average of 61.90±32.12) (Table 3).

**Table 3:** EORTC-QLQ-C30 scale's scores

EORTC-QLQ-C30 variables	Mean	SD	Minimum	Maximum
Global health status/Qol	50.00	21.73	16.67	83.33
**Functioning scales**				
Physical functioning	54.60	27.85	0.00	93.33
Role functioning	69.04	31.30	0.00	100.00
Emotional functioning	56.34	31.94	0.00	100.00
Cognitive functioning	72.22	31.32	0.00	100.00
Social functioning	75.39	17.96	33.33	100.00
**Symptom sacles**				
Fatigue (FA)	61.90	31.40	0.00	100.00
Nausea and vomiting (NV)	45.23	35.01	0.00	100.00
Pain (PA)	40.47	29.61	0.00	100.00
Dyspnoea (DY)	36.50	31.45	0.00	100.00
Insomnia (SL)	41.26	37.86	0.00	100.00
Appetite loss (AP)	46.03	34.11	0.00	100.00
Constipation (CO)	44.44	79.11	0.00	333.33
Diarrhoea (DI)	28.57	42.53	0.00	100.00
Financial difficulties (FI)	61.90	32.12	0.00	100.00

## Discussion

The purpose of our study is to investigate health related quality of life in 21 male patients affected with breast cancer and the clinico-pathological features over a period of 6 years at the university hospital of Casablanca-Morocco. Our study is the first in our country to assess quality of life (QoL) in male patients affected with breast cancer. According to literature, men tend to be diagnosed at an older age than women. The majority of our patients were in the sixth to eighth decade of their life, the mean age was 67.3 years; 17.7 months was the average consultation delay among our patients, delayed presentation was mainly related to a lack of education and poor socio-economic background in our country. Ninety-five point three percent of patients presented with infiltrating ductal carcinoma (IDC). As reported in the literature, IDC is the most commonly encountered histopathology [10-12]. Most authors prefer modified radical mastectomy for surgical excision of these cancers, and some suggest simple mastectomy followed by adjuvant treatment [13-15]. Surgery was the preferred procedure, in our study; all patients underwent surgery, while chemotherapy, radiotherapy, and hormone therapy were used in the adjuvant setting. Due to the high positivity of hormone receptors in male breast cancer (83%), most cases received hormone therapy. This has been shown to lead to increased survival rates in patients with hormone-sensitive disease and today is generally considered the standard adjuvant treatment for hormone-dependent male breast cancer [16]; also, hormone therapy has been proven to help in metastatic disease in females and males [17-19]. The 3 most important prognostic factors are tumor size, lymphatic invasion and axillary node status. Unfortunately, nodal involvement was presented in up to 62% of our patients [13, 20, 21].

Generally, cancer patients have worse HRQoL compared to the general population. Increasing age is associated with worse HRQoL in physical function and constipation, while it is associated with better with HRQoL in social functioning, insomnia and financial problems [22], comparing to this study [22], our results match; the subs cores related to social functioning were the highest rated scores (75.39±17.96) and this can be explained by an effective social support system in our community which plays an important role in reducing pressure and improving health, and by the spiritual and religious context characterized by solidarity and support. On the other hand, physical function was the less rated score in our study (54.60±27.85) and fatigue was the most common symptom, this was due to the elderly study population. Males had positive emotional functioning in our study (56.34±31.94), compared to females with breast cancer, who had poor emotional functioning. This may be related to the fact that women have to deal not only with the trauma of deformity, but also with the fear of rejection by their partners and loss of femininity [23]. Furthermore, overall QoL appeared to improve after treatment, and this improvement was seen in global health status, functional scales and symptom report scales.

## Conclusion

Quality of life appears to be better than expected and it generally improves after treatment. Further, emotional and social functioning are well rated, even in the absence of corresponding increases in physical functioning, and this is explained by and effective social support system in our community. As for prevention, public education should be oriented toward men at higher risk in order to reduce the time between the onset of symptoms and consultation. In addition, issues related to the disease, side effects of treatment and sexual functioning should receive more attention when studying quality of life in breast cancer male patients.
